# Understanding how to live with hepatitis B: a qualitative investigation of peer advice for Chinese people living with hepatitis B in Australia

**DOI:** 10.1186/s12889-022-12907-5

**Published:** 2022-03-18

**Authors:** Jack Wallace, Yinzong Xiao, Jess Howell, Alex Thompson, Nicole Allard, Emily Adamson, Jacqui Richmond, Behzad Hajarizadeh, Melanie Eagle, Joseph Doyle, Margaret Hellard

**Affiliations:** 1 Burnet Institute, Melbourne, VIC 3004 Australia; 2 La Trobe University, Bundoora, VIC 3086 Australia; 3 Centre for Social Research in Health, University of New South Wales Sydney, Kensington, NSW 2052 Australia; 4 Department of Gastroenterology, St Vincent’s Hospital, Fitzroy, VIC 3065 Australia; 5 University of Melbourne, Parkville, VIC 3010 Australia; 6 School of Public Health and Preventive Medicine, Monash University, Melbourne, VIC 3004 Australia; 7 World Health Organization Collaborating Centre for Viral Hepatitis, The Doherty Institute, Melbourne, VIC 3004 Australia; 8 Kirby Institute, University of New South Wales Sydney, Kensington, NSW 2052 Australia; 9 Hepatitis Victoria, Brunswick, VIC 3056 Australia; 10 Department of Infectious Diseases, The Alfred and Monash University, Melbourne, VIC 3004 Australia

**Keywords:** Hepatitis B virus, Health beliefs, China, Diagnosis, Health information

## Abstract

**Background:**

Hepatitis B is a chronic viral infection, a leading cause of primary liver cancer and identified as a major public health priority by the World Health Organization. Despite a high proportion of people in Australia who have been diagnosed with hepatitis B, significant gaps remain in health care access and in accurate knowledge about hepatitis B. Most people with hepatitis B in Australia were born in China, where the infection has an intergenerational impact with significant social implications resulting from the infection. Understanding how people of Chinese ethnicity with hepatitis B understand and respond to hepatitis B is imperative for reducing morbidity, mortality, and the personal and social impact of the infection.

**Methods:**

Qualitative semi-structured interviews with people with hepatitis B of Chinese ethnicity recruited through a specialist service identified the advice people with hepatitis B thought was important enough to inform the experience of people newly diagnosed with hepatitis B. A thematic analysis of the data privileged the lived experience of participants and their personal, rather than clinical, explanations of the virus.

**Results:**

Hepatitis B infection had psychological and physical consequences that were informed by cultural norms, and to which people had responded to with significant behavioural change. Despite this cohort being engaged with specialist clinical services with access to the most recent, comprehensive, and expert information, much of the advice people with hepatitis B identified as important for living with hepatitis B was not based on biomedical understandings. Key suggestions from people with hepatitis B were to form sustainable clinical relationships, develop emotional resilience, make dietary changes, regulate energy, and issues related to disclosure.

**Conclusions:**

The study highlights conflicts between biomedical and public health explanations and the lived experience of hepatitis B among people of Chinese ethnicity in Australia. Beliefs about hepatitis B are embedded within cultural understandings of health that can conflict with bio-medical explanations of the infection. Acknowledging these perspectives provides for insightful communication between health services and their clients, and the development of nuanced models of care informed by the experience of people with hepatitis B.

## Key points for decision makers

- Hepatitis B is a public health priority given increasing deaths resulting from the infection. Chinese born people are the primary population affected by hepatitis B both within their country of origin and, resulting from migration, within many other countries.
- Within Chinese communities, hepatitis B has significant social implications within a population who lack knowledge, particularly biomedical explanations, of this largely asymptomatic infection.
- The explanations and descriptions of hepatitis B provided by Chinese people living with hepatitis B are embedded within cultural understandings of health, and these can conflict with bio-medical explanations of the infection. Understanding and responding to these conflicts can provide a more nuanced and persuasive explanation of the infection for people with hepatitis B.

## Background

Hepatitis B is a chronic viral infection affecting the liver; without treatment, 25% of people with chronic hepatitis B develop advanced liver disease such as cirrhosis over 5–8 years with up to 5% developing liver cancer annually [1]. Globally, hepatitis B is a leading cause of primary liver cancer [2]. In 2016, the World Health Organization established targets calling for the elimination of viral hepatitis as a public health threat by 2030 [3], and hepatitis B has been identified as a major public health priority by Australian federal and state governments [4, 5].

At a global level, 10.5% of the estimated 257 million people with hepatitis B are diagnosed, with only 5% of people with hepatitis B who are eligible and require anti-viral treatment receiving treatment [6]. In Australia, an estimated 32% of people with hepatitis B are undiagnosed, making them vulnerable to liver disease and cancer. Despite the relatively high proportion of people diagnosed in Australia compared to many countries globally, this has not translated into increased engagement in clinical care, with only 22% of people diagnosed with hepatitis B engaged in clinical care, and less than one third of people eligible for treatment currently receiving antiviral treatment [7]. Current hepatitis B models of care respond to the biomedical aspects of the infection, neglecting the psychosocial or psychological impact of the infection [8].

The largest population affected by hepatitis B in Australia are people born in China [9], where hepatitis B transmission primarily occurs from mother to child, and where one in ten people over the age of 20 years live with hepatitis B. Studies have revealed hepatitis B affecting education and employment choices, economic opportunities, and the development of intimate relationships in China [10–13]. These social implications are further exacerbated with the marginalisation of people with hepatitis B from stigma, self-stigma, and discrimination [10, 11, 14–18]. Given the substantial size of the Chinese diaspora, and numbers of people of Chinese ethnicity living with hepatitis B in Australia and in other similar countries, particularly understanding how this population comprehend and respond to hepatitis B is imperative for reducing morbidity, mortality, and of the personal and social impact of the infection [4]. Systematic deficits in how people with hepatitis B are diagnosed provide the context where significant gaps in accurate biomedical knowledge of people with hepatitis B of their infection have been identified [19, 20].

Given these gaps, this study sought to identify what and how people understood their hepatitis B infection by asking people with hepatitis B to describe the infection and what advice they would give about living with hepatitis B to someone who was newly diagnosed. This exploratory investigation is unique by describing hepatitis B based on lived experience, rather than using just biomedical descriptions. Identifying an explanatory model for hepatitis B based on the lived experience of people with hepatitis B provides the opportunity to combine this narrative with biomedical interpretations of hepatitis B. This combination can support developing nuanced and sustainable clinical experiences through improved communication that is grounded in the everyday lives of people with hepatitis B.

## Methods

Interviews were held with 30 Mandarin and/or English-speaking adults of Chinese ethnicity with hepatitis B recruited through one specialist clinical service (St Vincent’s Hospital, Melbourne). Chinese ethnicity was self-described, with participants born in mainland China (*n* = 19), Malaysia (*n* = 5), Taiwan (*n* = 2), Indonesia (*n* = 1), Vietnam (*n* = 1), and undisclosed (*n* = 2). Most interviews (28) were conducted in person in a private room located at the clinic, with two interviews conducted by telephone.

All patients meeting the study criteria attending their liver clinic appointment during the study period were referred by several specialist physicians to the researchers (the first two authors), and then provided with written information in English and simplified Chinese describing the study. Interviews lasted between 15 and 30 min in duration and were conducted with either one or two researchers: one English speaking, and one fluent in English and Mandarin. Twelve interviews were held in English, two in a mixture of English and Chinese, and 16 in Chinese. Interviews were electronically recorded, and transcribed in English or Mandarin, with the Chinese transcripts translated into English. The sample size was determined by data saturation, with no new themes arising after 30 interviews.

Coding and analysis were independently conducted by the first two authors using the thematic analysis stages described by Braun and Clarke [21]. Transcripts were independently manually coded and entered into NVivo 12 (QSR International Pty Ltd., Vic, Australia) software after careful reading and familiarising of individual transcripts. An agreed analytical framework was refined after identifying, reviewing and comparing themes across the data set.

An interview guide was used to direct the interviews and is found as Table 1. The interview guide sought information on:

- When and how people were diagnosed
- If having hepatitis B changed a participant’s life, and the impact of these changes.
- What the participant would tell their best friend, if their friend had been diagnosed with hepatitis B.
- The information that could have helped the participant when they were diagnosed with hepatitis B, and where they obtained this information?

**Table 1:** Interview Guide

Information sought	Interview Prompts
When and how people were diagnosed	• Who told you that you had hepatitis B? • When did you find out that you had hepatitis B? • How old were you when you were told you had hepatitis B? • Were you tested for hepatitis B by a doctor, or someone else? • What information were you given when you were told that you had hepatitis B? • Do other people in your family have hepatitis B?
Has having hepatitis B changed a participant’s life, and if this has occurred in a negative and/or positive way.	• Has having hepatitis B changed your life? How? Have there been good things that have happened because you have hepatitis B?
What the participant would tell their best friend, if their friend had been diagnosed with hepatitis B.	• You’ve told us how you were diagnosed and the information that you were given. • From your experience, what have been the 5 most useful pieces of information that you would tell your best friend or a family member about living with hepatitis B? • What information would have been the most useful when you were told you had hepatitis B?
Identifying the information that would have helped the participant the most when they found out they had hepatitis B. Where did they get this information?	• Where did you get this information? And why was it useful for you?
Any suggestions from the participants for interventions to increase the number of people with hepatitis B being diagnosed.	• There are many people who have hepatitis B that don’t know that they have the virus. From your experience, what could you suggest would increase the number of people with hepatitis B being diagnosed?

Participation in the research was voluntary, with all participants providing verbal consent that was electronically recorded prior to the commencement of the interview. No participant refused to participate once approached by the researchers. Ethical approval for the study was obtained from the Alfred Hospital Human Research Ethics Committee (537/18) with Governance Approval from St Vincent’s Hospital (Melbourne) (044/19).

## Results

Data was collected from 14 women and 16 men with an age range between 29 and 71 years, who had lived in Australia for between two and over 20 years. The median age of participants was 49, and the interquartile range was 38–63 years. Most participants had significant lived experience of hepatitis B with 18 participants being diagnosed with hepatitis B for over 20 years, and a further 10 participants diagnosed between 10 and 20 years ago. Twenty-two participants reported an intergenerational impact of hepatitis B having a family member such as a parent, sibling or significant other living with hepatitis B.

There were a broad range of circumstances in which participants were tested and diagnosed. The single most common location for being tested for hepatitis B was through a health service (*N* = 23), while five were diagnosed through school-based testing, and another two through workplace-based testing. Most participants (*N* = 19) were currently receiving pharmaceutical treatment. Most participants were diagnosed in China (*N* = 16) or Australia (*N* = 9), with the remainder diagnosed in Malaysia (*N* = 4) and New Zealand (*N* = 1).

Given the well-reported lack of systematic information provided to people with hepatitis B about the infection at the point when people are diagnosed [19, 20] and the resulting incomplete biomedical knowledge about hepatitis B [8, 22–27], a broad range of assumptions about the aetiology of the infection were described. While not directly relating to the aim of the study, these health beliefs formed the context for some of the advice provided to peers. Understanding hepatitis B occurring as a result of a progression of unrelated health conditions was described by one participant:

*Rheumatoid arthritis, then it became an immune system disorder, and then it became ... getting hepatitis C and hepatitis B, like this* (Interview 21).

Instead of accurate understandings of hepatitis B exposure occurring through infection at birth, or exposure through inadequate health service infection control procedures, hepatitis B was understood as a genetic condition, *it’s like gene transfer to me* (Interview 15). Even when accurate information was provided by clinicians, the information was understood in ways that possibly conflicted with the intent of the information:

*I … listened to the doctor to explain again. They explained very patiently, then I started to understand, ‘oh this is genetically inherited’* (Interview 27).

Other understandings included hepatitis B as a food or food utensil borne pathogen: “*it must be spread via food, also... sharing the tableware, this is inseparable. Because in China, …everyone is eating together”* (Interview 10), or alternatively resulting from bingeing with food and alcohol, with one participant noting:

*When I went back to China, all our classmates were very happy … everyone ate together … so happy that we feasted until we fell down … that’s probably the reason.* (Interview 24).

Several methods for assumed transmission of hepatitis B were described, including being infected through the workplace from welding machine fumes and “*the smell of burning*” (Interview 12), or where infection resulted from *“the big environment of where we from, in my age group, many of them are hepatitis B carrier*” (Interview 28). For one-person, physical closeness was key in their understanding of hepatitis B exposure:

*I like keeping the distance just like between you and me. …if people talk too close to me, I would say that I might have this hepatitis B, … I would say it’s fully controlled, but we still (need to) keep a distance* (Interview 18).

While assumptions of hepatitis B aetiology, exposure and transmission varied, there were five key messages people thought were important that a newly diagnosed person needed to know about living with hepatitis B:

1. Maintain a relationship with clinical services
2. Diet
3. Regulate energy levels
4. Resilience
5. Disclosure

### Maintain a relationship with clinical services

The most commonly reported piece of health advice for people newly diagnosed with hepatitis B was for people to establish a relationship with a medical professional, maintain access with clinical management, and to be regularly clinically monitored:

*Professional advice is the most important thing* (Interview 2).

*Just do the regular check-up, just to monitor your liver* (Interview 4).

Current international guidelines recommend anti-viral treatment for patients at risk of liver cirrhosis and/or liver cancer. For the vast majority of people with hepatitis B, the primary form of clinical engagement recommended is for six-monthly blood tests to monitor for indications of liver disease progression, or in some cases a six-monthly ultrasound for liver cancer surveillance [28]. One participant was clear and accurate in their understanding for the rationale for being clinical monitored:

*The most important thing is to do the liver function tests … if the reading is fine, (the liver) should be fine. If the reading is not … see a doctor. What I know is that the liver function test, if that value get too high and maintain for too long, it will hurt our liver and I could … have liver cancer* (Interview 19).

Clinical staff were identified as the primary and authoritative source for accurate information, “*get more information from your doctor”* (Interview 9*),* with differences described in the availability of information about hepatitis B between Chinese and Australia online sources:

*We could Google it here ... But in China, if you use Baidu (a Chinese-based search engine) to search, it would... scare you to death. The top 100 probably are all selling medicine* (Interview 10).

Nineteen participants were currently receiving pharmaceutical treatment for their infection with several participants recommending that people who are newly diagnosed, also access treatment:

*Take the test, tablets, should be fine and make sure you be at the doctor every 6 months* (Interview 5).

There is a risk of developing viral resistance if prescribed medicine is not used as stipulated. One participant used a Chinese colloquialism to describe persistence and perseverance in the regular use of prescribed treatment:

*If you take this medicine, don't forget to take it... don’t go fishing for three days and dry the nets for two* (Interview 18).

### Diet

Eight participants reported their possible exposure, or their understanding of hepatitis B transmission occurring through eating food or eating outside of the home:

*This disease is spread by eating. If you have this disease in your food, you will be infected* (Interview 17).

*My family doctor ... says it’s very easy to catch it even in dining room, or even in water, maybe somebody else pass to you, easy, because of food…because of chopsticks, anything could happen* (Interview 22).

This incorrect transmission information for some participants resulted in applying unnecessary prevention strategies. The following quotes highlight both the level of vigilance experienced by one participant, and another who recognised while this vigilance was unnecessary, there was still underlying assumptions of a risk involved in sharing food utensils.

*I have to mind my chopsticks both at home and outside... I don’t eat like other people ... the bad things are passed on to others... I can't go on my conscience* (Interview 11).

*If friends invite to have meals, I will go, but I would request public chopsticks … now … the medical techniques and conditions are very good, but, just in case, I use public chopsticks* (Interview 18).

While incorrectly seen as a possible route of exposure for several participants, food consumption had a role as an important health promotion message, with various suggestions for maintaining a good diet including balanced eating including “cool vegetables”, and consuming “light” food which was food that was not oily, spicy or strongly flavoured:

*The general rule is that most of the times disease goes in by the mouth, (and you need to) eat more food that cools your body or similar, to…to clean up the hot things inside (your body)* (Interview 12).

Most participants noted the need for people with hepatitis B to reduce or stop alcohol use, although this strategy had both positive and negative implications

*I can’t take alcohol at all. And that’s a bit hard for me, because in work sometimes I have to have some social drinks, but I try to avoid things like that* (Interview 18).

But the issue of alcohol use, particularly within the Australian context where harmful levels of alcohol use are a major health issue [29], and where its use is entwined with many social and cultural obligations, was one of several circumstances in which people with hepatitis B felt obliged to disclose their infection:

*Sometimes I say to my friends I’m not drinking or I’m reducing my drinking because I have this hepatitis B, and sometimes I even use it to be an excuse to be honest (laugh), ‘don’t force me to drink, it’s health related’* (Interview 13).

*Generally, if someone is curious about why I don’t drink in a social setting, and if I think that it’s kind of ok, then I will tell them* (Interview 8).

### Regulate energy level

A key feature of the advice, and one which could have a significant economic and psychological impact on people with hepatitis B and their families was an understanding that resting or not working hard was an intervention that could protect their health. For one participant conserving energy was seen as being more important than medicine:

*Rest, I think it is more important than drugs* (Interview 10).

Within a context of a lack of more accurate knowledge, the experience of over-exertion was also seen to have a significant impact on liver disease progression.

*Don’t do those (heavy) work. and you can’t do those overloaded work either...My son relapsed for the second time was because… he was doing the fitness training every day* (Interview 25).

Conserving bodily energy was seen to have a virological impact in a group of people who were confident in their knowledge that a low viral load was seen to be a “good thing”

*Sometime probably tired, there will be like, the virus will be like up a little bit and you know, sometimes you relaxing more and then they will be like down* (Interview 15).

### Resilience

One key theme in suggestions for people who have been newly diagnosed relates to the need for developing psychological resilience. The need for psychological support was noted by one participant, particularly given the lifelong nature of the infection:

*You have to be prepared for this idea, this disease may accompany you for a lifetime, it will have some impact on you ... it had a big impact on me. If you let me make a suggestion ...to have a psychological counselling* (Interview 10).

The need for a person newly diagnosed with hepatitis B to respond to the development of a sense of resignation or fatalism in accepting the need to live with hepatitis B was expressed by several participants:

*If you (are) frowning at life, you have the disease, if you don’t frown, you also have the disease, right?* (Interview 12).

As reflected in the previous quote, living with a largely asymptomatic infection was highlighted as being intrinsically stressful as a result of living with a chronic infection, fear of onward transmission or of the social implications of the infection. The experience of uncertainty related to living with hepatitis B was seen as unique to those people living with the infection and reflected an essential difference between people living with and those without the virus.

*People without hepatitis B won’t have pressure, people who have hepatitis B like us are having pressure, right? You don't know what will happen in the future* (Interview 11).

### Disclosure

A broad range of responses were received from participants when asked what advice they would give to people who had been newly diagnosed about disclosing their infection. Several people described that as hepatitis B was not casually transmitted, or that the person with hepatitis B was on treatment and therefore had little viral load, there was no need for them to disclose their infection to people outside of their family:

*I don't think it's necessary to tell others because the doctor also told us that if you take this medicine, you will not transmit (the virus) to others, so there is no need to tell others* (Interview 25).

The experience for people who were born and diagnosed in China, affected their behaviour in Australia. In relation to disclosure, this meant risking social isolation as a result of the marginalisation related to hepatitis B if they disclosed, alongside a Chinese cultural norm of not discussing health outside of immediate family or as a part of general conversation:

*In our country, we don’t tell people these things ... People might think like where did you get this, whether you are going to pass this to me, so I might lose all my friends ... but I don’t tell them* (Interview 6).

There were also significant differences in terms of privacy and the broad impact that hepatitis B has in China in comparison to Australia:

*People treat this as individual privacy, which is different from what’s in China, you have to do the test whenever you have to enter a school or work, and everyone knows* (Interview 2).

While most participants were clear in their advice to limit disclosure, two participants, both born in Malaysia, were clear in having no reservations in disclosing their hepatitis B infection:

*I’m very open about it to be honest ...I just don’t feel like it should be something I need to hide* (Interview 13)

## Discussion

This study investigated the information people with hepatitis B thought was important enough to inform the experience of others with hepatitis B, particularly people who had been newly diagnosed. Key pieces of advice about hepatitis B were not based on biomedical understandings despite this cohort being highly engaged with specialist clinical services, and having access to the most recent, comprehensive, and expert information. The study highlights essential conflicts between the lived experience of hepatitis B among Chinese people in Australia, with biomedical and public health descriptions and expectations of the infection.

While effective interventions included disclosing their infection to their immediate family and ensuring their family were immunised, several participants sought to further reduce possible exposure by not sharing food or eating utensils, even when the participant recognised this as unnecessary and stressful. Observing and responding to hepatitis B as a purely biomedical condition that affects the liver ignores the substantial and relevant psychological, mental and emotional impact upon individuals and their households and the need to develop systematic health system responses to these understandings [8].

The role of food in hepatitis B care, including what, how and where food is consumed was significant and participants variously identified food as both a source of exposure and method of transmission, with consumption of the ‘right’ food and having a balanced diet were seen to protect the liver and be a key factor in maintaining health. While these reflect traditional Chinese medical understandings of the need to balance energy within the body, they have little relationship to those expounded by biomedicine. The messages from participants about alcohol were clear and unequivocal with significant social and work impacts given the role of alcohol within Australian culture.

Hepatitis B is well characterised in China as having a substantial impact through the social marginalisation of people infected with the virus [10]. This marginalisation is only possible after disclosure of the infection. As noted by the need for information to reduce the psychological impact of the infection, knowing accurate transmission routes for hepatitis B reduced the need for widespread disclosure of the infection and reduced the possible stress associated with this disclosure. This limiting of disclosure is particularly important for people with viral hepatitis in China, while for others the lack of prevalence and profile in Australia reduced any obligation people felt for disclosing.

There is a well-documented need to respond to the psychological needs of people with hepatitis B, with studies showing a significant psychological impact [12, 22, 30]. Within this sample, the psychological pressures were evident as a result of living with a chronic and lifelong infection where no cure is currently available, while for others it related to the spectre of infection with a virus that for many had caused significant grief and loss within family networks. In addition, and in reflection of the interactive nature of people’s responses, participants described other hepatitis B-related stressors included seeking to reduce hepatitis B exposure to others through a number of interventions, most of which would have little or no impact, and in spite of being aware of hepatitis B vaccination.

Participants in this study were a cohort of patients within one specialist liver clinic, and this essentially limits the scope of the data collected and its implications. The rationale for recruiting through a specialist service was to identify key issues for people who were engaged in their clinical management of hepatitis B, and who would be seen as people who have successfully engaged with their infection on a long term and consistent basis. There are limits to extrapolating the data considering that only a minority of people with hepatitis B at a global level have been diagnosed and know of their infection, let alone regularly access specialist care.

## Conclusion

Complex hepatitis B testing protocols and health advice have been developed by public health and clinical specialists, without adequate or systematic input from people with hepatitis B, who report significant deficits in how they are diagnosed. While not wanting to reinforce non-scientific perspectives about hepatitis B infection, particularly given the lack of, or incorrect information about the infection, acknowledging these perspectives could provide for the development of stronger, more insightful, and nuanced communication with health service staff based on the experience of people living with the infection. The findings provide a rationale for recognising the important clinical role played by people with hepatitis B. There continues to be a need for a program of sustainable and systematic provision of information with the possible use of peer-based clinical navigation for people with hepatitis B.

## Data Availability

Data generated during the study are not publicly available for confidentiality reasons and may be available from the corresponding author on reasonable request.
